# Acute Chikungunya Virus Infection Triggers a Diverse Range of T Helper Lymphocyte Profiles

**DOI:** 10.3390/v16091387

**Published:** 2024-08-30

**Authors:** Ramayana Morais de Medeiros Brito, Marília Farias de Melo, José Veríssimo Fernandes, Joanna Gardel Valverde, Paulo Marcos Matta Guedes, Josélio Maria Galvão de Araújo, Manuela Sales Lima Nascimento

**Affiliations:** 1 Department of Parasitology, Institute of Biological Sciences, Federal University of Minas Gerais, Belo Horizonte 31270-901, Brazil; 2 Department of Microbiology and Parasitology, Biosciences Center, Federal University of Rio Grande do Norte, Natal 59078-970, Brazil; 3 Institute of Tropical Medicine of Rio Grande do Norte, Federal University of Rio Grande do Norte, Natal 59078-970, Brazil; valverdejoanna@gmail.com

**Keywords:** Chikungunya virus, adaptive immune response, anti-viral response

## Abstract

Chikungunya virus (CHIKV) is an arbovirus causing acute febrile illness with severe joint pain, often leading to chronic arthralgia. This study investigated the adaptive immune responses during the early stages of symptomatic acute CHIKV infection, focusing on the transcription factors and cytokines linked to Th1, Th2, Th17, and Treg cells. Thirty-six individuals were enrolled: nine healthy controls and 27 CHIKV-positive patients confirmed by qRT-PCR. Blood samples were analyzed for the mRNA expression of transcription factors (Tbet, GATA3, FoxP3, STAT3, RORγt) and cytokines (IFN-γ, IL-4, IL-17, IL-22, TGF-β, IL-10). The results showed the significant upregulation of Tbet, GATA3, FoxP3, STAT3, and RORγt in CHIKV-positive patients, with RORγt displaying the highest increase. Correspondingly, cytokines IFN-γ, IL-4, IL-17, and IL-22 were upregulated, while TGF-β was downregulated. Principal component analysis (PCA) confirmed the distinct immune profiles between CHIKV-positive and healthy individuals. A correlation analysis indicated that higher Tbet expression correlated with a lower viral load, whereas FoxP3 and TGF-β were associated with higher viral loads. Our study sheds light on the intricate immune responses during acute CHIKV infection, characterized by a mixed Th1, Th2, Th17, and Treg response profile. These results emphasize the complex interplay between different adaptive immune responses and how they may contribute to the pathogenesis of Chikungunya fever.

## 1. Introduction

Chikungunya virus (CHIKV) is a re-emerging alphavirus responsible for outbreaks of acute febrile illness characterized by severe and often debilitating joint pain, which can progress to chronic arthralgia in some individuals [1]. First identified in Tanzania in 1952, CHIKV has since caused numerous epidemics worldwide, with significant public health impacts, particularly in tropical and subtropical regions [2]. The virus is an arbovirus primarily transmitted by *Aedes* mosquitoes, including *Aedes aegypti* and *A. albopictus*, which are also vectors for dengue, Zika, and yellow fever virus [3,4,5,6].

The adaptive immune responses against arboviruses, such as dengue virus (DENV) and Zika virus (ZIKV), provide valuable insights into the immune dynamics during CHIKV infection. Dengue virus infection elicits strong Th1 responses, characterized by the expression of T-bet as the main transcription factor and high levels of IFN-γ, which are crucial in controlling viral replication. However, secondary DENV infections can lead to severe dengue, partly due to antibody-dependent enhancement (ADE) and dysregulated T cell responses [7]. Th2 responses, marked by GATA3 expression and IL-4 and IL-10 production, have also been implicated in modulating the severity of dengue [8]. Similarly, Zika virus infection induces a robust immune response, with the early production of type I interferons playing a critical role in controlling viral replication. The role of Th1 and Th17 responses has been highlighted, with evidence suggesting that an effective Th1 response is protective, while Th17 responses, marked by the expression of RORγt and production of IL-17, might contribute to neuroinflammation and fetal damage in congenital Zika syndrome [9]. Regulatory T cells (Tregs), represented by the expression of FoxP3 as the major transcription factor, are also involved in ZIKV infections, potentially mitigating the immunopathology by controlling excessive immune activation [10].

CHIKV infection has been shown to induce a broad and dynamic immune response. During the acute phase of infection, there is the upregulation of innate immunity-related transcription factors, such as TLR3, RIG-1, and MDA5, followed by the induction of pro-inflammatory and antiviral cytokines such as IFN-α, IL-6, and TNF-α, which are crucial for initial viral control [11,12]. However, the persistent activation of these pathways can lead to chronic inflammation and joint damage, highlighting the dual role of the immune response in both protection and pathogenesis [13]. The adaptive immune response, particularly the T cell response, is essential for long-term immunity and viral clearance [14]. Recent studies have indicated that CHIKV infection triggers a complex interplay between different Th cell subsets. Increased levels of Th1 and Th17 cytokines have been observed in patients with acute CHIKV infection, suggesting a mixed Th1/Th17 response [15,16,17]. Moreover, the role of Tregs during CHIKV infection is still being elucidated, with some studies suggesting that Treg responses might modulate the severity of the disease by controlling excessive inflammation [18,19].

Given the critical roles of these Th cell subsets in modulating the immune response to CHIKV, understanding their specific contributions and regulatory mechanisms is essential. This knowledge could lead to the development of targeted therapeutic strategies aimed at modulating the immune response to achieve better clinical outcomes for CHIKV patients. This study aims to provide a comprehensive analysis of the adaptive immune response’s main markers in the early stages of symptomatic acute CHIKV infection, with a focus on the expression of key transcription factors and cytokines associated with Th1, Th2, Th17, and Treg cells. By elucidating these immune response profiles, we seek to enhance our understanding of the immunopathogenesis of Chikungunya fever and inform the development of potential therapeutic interventions.

## 2. Materials and Methods

### 2.1. Ethical Statement and Study Design

The study followed the norms issued by the Research Ethics Committee, Brazilian Ministry of Health, and following the Helsinki Declaration, certified by the National System of Ethics in Research, under protocol CAAE:51057015.5.0000.5537.

Thirty-six individuals were enrolled in the study, comprising 9 healthy subjects (control group) and 27 patients with a positive qRT-PCR for CHIKV. The CHIKV-positive individuals enrolled in this study were 40.7% male and 59.2% female; they had a mean age of 39.3 (±18.45) years old and CT values of 27.95 (±4.92). Blood samples were collected within five days after the beginning of the symptoms in local basic health units and private and/or public hospitals in the Municipality of Natal, State of Rio Grande do Norte, Northeastern Brazil, in the period from 2015 to 2016. All participants were over 18 years old and were adequately informed about the research and signed a consent form.

### 2.2. Blood Collection and Processing

Peripheral blood samples were collected using a vacuum collection system (Vacutainer^®^, BD Biosciences, San Jose, CA, USA) and processed to obtain peripheral blood mononuclear cells (PBMC) through a concentration gradient using Ficoll-Hypaque solution (Sigma-Aldrich, St. Louis, MO, USA). After isolating the PBMCs, the cells were stored in TRIzol (Invitrogen™, Carlsbad, CA, USA) aliquots at −20 °C for subsequent analysis.

### 2.3. Viral RNA Extraction and Detection of CHIKV by qRT-PCR

Viral RNA was extracted from the patients’ peripheral blood samples using the QIAmp Viral RNA Mini Kit (Qiagen, Germantown, MD, USA), following the manufacturer’s instructions. The quantification of the CHIKV load was carried out by qRT-PCR, as previously described [20]. To detect the viral RNA, the probes used were CHIKV 6919P (Applied Biosystems^TM^, San Francisco, CA, USA) and the primers were CHIKV 6856F and CHIKV 6981R (Invitrogen™, Carlsbad, CA, USA), as described in Table 1.

### 2.4. Total mRNA Extraction, cDNA Synthesis, and qRT-PCR

Total RNA was extracted using the TRIzol^®^ reagent (Invitrogen^TM^, Carlsbad, CA, USA) and the SV Total RNA Isolation System (Promega, Madison, WI, USA), following the manufacturers’ instructions. The cDNA was then synthesized using the High Capacity cDNA Reverse Transcription Kit (Applied Biosystems^TM^, San Francisco, CA, USA), following the manufacturer’s instructions. 

The mRNA expression levels for the transcription factors (FoxP3, STAT3, RORγt, GATA3, Tbet, and PU.1), iNOS, AHR, and cytokines (IFN-y, IL-17, IL-4, IL-9, IL-22, TGF-b, and IL-10) were determined in the PBMC samples by qRT-PCR using the primers described in Table 2. The qRT-PCR was performed using the SYBR Green Master Mix system and carried out in a 7500 Fast Real-Time thermal cycler (Applied Biosystems^TM^, San Francisco, CA, USA). The standard cycles were 2 min at 50 °C, 10 min at 95 °C, and 40 cycles of 15 s at 95 °C, followed by 1 min at 56 °C. The final cycle was 20 min at increasing temperatures from 60 to 95 °C to obtain the transcript dissociation curve, used to analyze the specificity of amplification. The mRNA expression levels were determined as the means of the CT values to calculate the relative expression in CHIKV-positive individuals compared to the healthy control group. All data were normalized to the housekeeping β-actin gene using the 2^(−ΔΔCT)^ method. Each sample was analyzed in triplicate.

### 2.5. Statistical Analysis

All data were analyzed using GraphPad Prism v. 8.0 (GraphPad, San Diego, CA, USA). To assess data normality, the Shapiro–Wilk test was performed. Non-parametric data were analyzed using the Mann–Whitney test, and outlier values were removed using the ROUT test. With non-parametric data, the Spearman correlation test was performed on the expression levels of the analyzed targets. Principal component analysis (PCA) was performed to verify the clustering pattern within the groups using the ClustVis tool [21]. Differences of *p* ≤ 0.05 were considered statistically significant and data are reported as boxplot graphs showing the median, with the data distribution displaying the maximum and minimum values.

## 3. Results

### 3.1. Symptomatic Acute Infection by CHIKV Induces a Mixture of T Helper Responses

**Figure 1:**
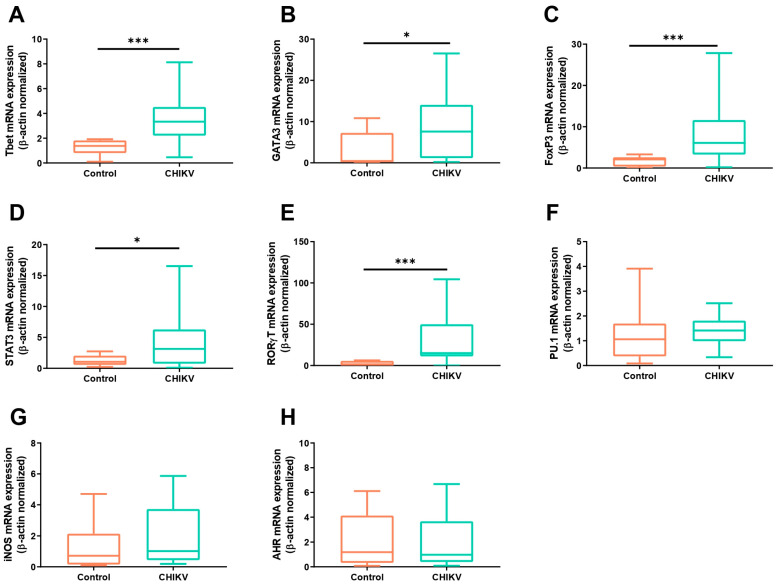
Expression levels of mRNA for the main transcription factors related to the Th1, Th2, Th17, and Treg immune responses. The mRNA expression for Tbet (**A**), GATA3 (**B**), FoxP3 (**C**), STAT3 (**D**), RORγT (**E**), PU.1 (**F**), iNOS (**G**), and AHR (**H**) in patients with acute CHIKV infection, compared to healthy individuals. The transcripts’ expression levels were normalized to the expression level of the β-actin housekeeping gene. * *p* < 0.05; *** *p* < 0.001.

In order to analyze the prevalence of the adaptive immune response profiles in the early stages of Chikungunya fever infection, we analyzed the mRNA expression of the main transcription factors related to Th1, Th2, Th17, and Treg immune responses in patients diagnosed with the viral infection. We observed that the expression of Tbet, GATA3, FoxP3, STAT3, and RORγt mRNAs was significantly upregulated during CHIKV infection, when compared to the healthy control group (Figure 1A–E). Interestingly, among all individuals, RORγt displayed the highest expression levels (RORγt—CHIKV patients: 15.07 ± 8.43; control group: 2.36 ± 0.9, *p* = 0.0004). No differences in the expression levels of transcripts for PU.1, iNOS, or AHR were found between the CHIKV-positive and control groups (Figure 1F–H).

**Figure 2:**
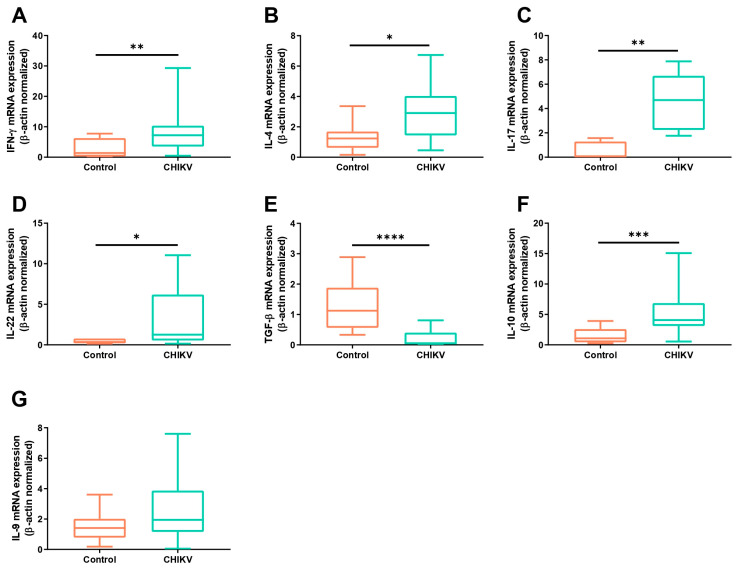
Expression levels of mRNA for the main cytokines related to the Th1, Th2, Th17, and Treg immune responses. The mRNA expression for IFN-γ (**A**), IL-4 (**B**), IL-17 (**C**), IL-22 (**D**), TGF-β (**E**), IL-10 (**F**), and IL-9 (**G**) in patients with acute CHIKV infection, compared to healthy individuals. The transcripts’ expression levels were normalized to the expression level of the β-actin housekeeping gene. * *p* < 0.05; ** *p* < 0.01; *** *p* < 0.001; **** *p* < 0.0001.

Regarding the mRNA expression for cytokines related to the T helper profiles, it was found that CHIKV-positive individuals showed significantly higher levels of IFN-γ, IL-4, IL-17, and IL-22 (Figure 2A–D). Interestingly, downregulated expression for TGF-β transcripts was found within CHIKV+ individuals (Figure 2E), while the IL-10 mRNA was found to be significantly upregulated when compared to the healthy controls (Figure 2F). No differences in the IL-9 mRNA expression were found between the patient and control groups (Figure 2G).

**Figure 3:**
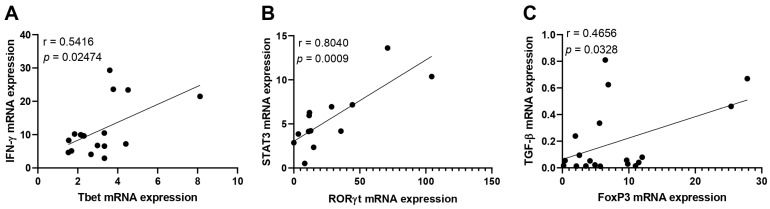
Correlation analysis between transcription factors related to Th1, Th17, and Treg immune responses. Correlation analysis between mRNA expression for IFN-γ and Tbet (**A**); STAT3 and RORγt (**B**); and FoxP3 and TGF-β (**C**) in patients with acute CHIKV infection, compared to healthy individuals. The analysis was performed using the Spearman correlation test.

The correlation analysis between the mRNA expression levels suggested that Tbet was upregulated together with IFN-γ (Figure 3A, r = 0.5416, *p* = 0.02477); RORγt was positively correlated with the expression of STAT3 (Figure 3B, r = 0.8040, *p* = 0.0009), representing the strongest correlation found, and FoxP3 was positively correlated with TGF-β (Figure 3C, r = 0.4656, *p* = 0.0328). 

Together, these data show that symptomatic acute CHIKV infection triggers a mixed immune response, marked by the upregulation of several signature transcription factors of distinct T helper cell populations: Tbet, GATA3, FoxP3, STAT3, and especially RORγt. Consequently, the corresponding cytokines, IFN-γ, IL-4, IL-10, IL-17, and IL-22, are also upregulated. These results suggest a complex mechanism of immune activation and regulatory imbalance during acute CHIKV infection.

### 3.2. Immunological Association with Viral Load during Acute CHIKV Infection

**Figure 4:**
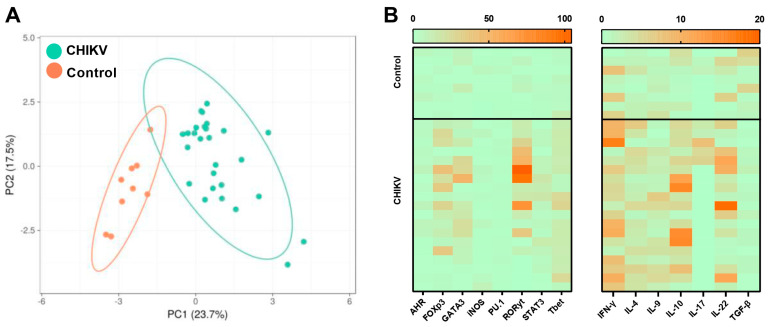
Clustering distribution of the CHIKV patients compared to healthy subjects. Principal component analysis highlighting the clustering patterns of CHIKV-positive patients (green) and healthy individuals (orange) (**A**). Heatmap evidencing the expression levels of the analyzed targets, compared with those of healthy subjects. The darker the orange color, the higher the mRNA expression level (**B**).

To analyze the clustering dynamics between CHIKV-positive individuals and healthy controls, we conducted principal component analysis (PCA). PCA is a statistical technique used to simplify the complexity in high-dimensional data while retaining trends and patterns. This analysis was used to distinguish between CHIKV-positive individuals and healthy controls based on the immunological markers analyzed here. The clear separation into distinct clusters (Figure 4A) indicates that the immune response profiles of positive individuals are significantly different from those of healthy controls. In summary, each point on the PCA plot corresponds to an individual’s immune response profile, with the positions along PC1 and PC2 reflecting the differences in the expression levels of the immune markers assessed. The distinct expression levels of the analyzed transcripts for AHD, FoxP3, RORγ, Tbet, iNOS, PU.1, GATA3, and STAT3 and for the cytokines IFN-γ, IL-4, IL-9, IL-10, IL-17, IL-22, and TGF-β likely highlight which immune markers might contribute most to the differences between the two groups (Figure 4B). The clear separation into distinct clusters highlights the altered expression levels of specific immune markers in CHIKV patients.

**Figure 5:**
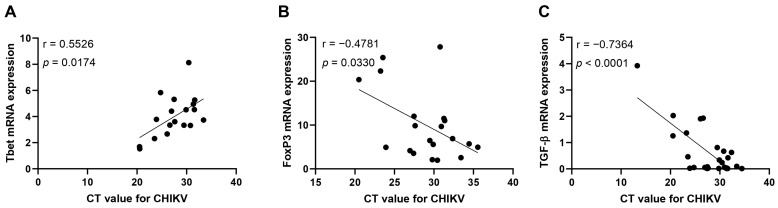
Interaction between viral load and mRNA expression level for immune-related markers. Correlation analysis between CT values and mRNA expression for Tbet (**A**), FoxP3 (**B**), and TGF-β (**C**) in patients with acute CHIKV infection, compared to healthy individuals. The analysis was performed using the Spearman correlation test.

To analyze the impact of the viral load on the immunological pathways studied, correlation analyses were performed and they revealed that the CT values were positively correlated with the mRNA expression levels for Tbet (Figure 5A). Considering that a higher CT means a lower viral load, these data indicate that the higher the Tbet expression, the lower the viral load. On the other hand, the CT values for the CHIKV viral load were negatively correlated with the mRNA expression levels for FoxP3 and TGF-β (Figure 5B,C), meaning that these markers are related to higher viral loads and host susceptibility. No correlations were found when analyzing the CT values against the mRNA expression of STAT3, RORγt, GATA3, IFN-γ, IL-4, or IL-10 (Figure S1).

These findings underscore the complex relationship between CHIKV infection, the viral load, and the immune response, pinpointing key markers represented by the increased expression levels of Tbet, GATA3, FoxP3, STAT3, and RORγt and the respective cytokines analyzed here, which differentiate affected individuals from healthy controls. The analysis reveals the Tbet pathway as key for viral clearance and FoxP3/TGF-β related to the viral burden.

## 4. Discussion

During the acute phase of CHIKV infection, the immune response is crucial in controlling the virus but can also contribute to the development of clinical symptoms [22]. The immune response mechanisms, while essential for viral clearance, are also associated with the severe joint and muscle pain characteristic of Chikungunya fever [23,24]. Understanding the balance between effective antiviral defense and the pathological consequences of immune activation is key to developing and improving therapeutic strategies to manage the disease [25]. In this sense, our study provides a comprehensive analysis of the main transcription factors and associated cytokines related to the adaptive immune response during acute CHIKV infection.

Herein, we observed the marked upregulation of transcription factors associated with Th1, Th2, Th17, and Treg cell subsets, indicating the complex interplay of these four profiles of adaptive immune responses. The polarized Th1 immune response is crucial for defense against intracellular pathogens, primarily through the production of IFN-γ, which activates macrophages and enhances the cytotoxic activity of natural killer (NK) cells and CD8+ T cells [26]. Although the role of IFN-γ induced during CHIKV infection remains unclear, the observed upregulation of Tbet and its correlation with IFN-γ in our study is consistent with previous research that has reported a robust Th1 response during the early stages of Chikungunya in both human disease and experimental murine models of CHIKV infection [15,27]. The significant increase in IFN-γ within individuals with acute CHIKV infection found in our study underscores the role of Th1 cells in controlling CHIKV infection, in which the increase in Tbet expression follows the decrease in the viral load.

On the other hand, the upregulation of GATA3 and IL-4 might suggest a concurrent Th2 response, which is typically associated with helminth infections and allergic reactions [28,29] and may contribute to the modulation of Th1/Th17 inflammatory reactions. The presence of both Th1 and Th2 responses simultaneously indicates a complex immune response, which may reflect the body’s attempt to counteract the virus while managing inflammation. 

It is important to mention that the pronounced upregulation of RORγt, alongside the increased expression of STAT3, highlights the role of Th17 cells during CHIKV infection, supported by the increased expression of the inflammatory cytokines IL-17, IL-6, IL-21, and IL-22, which are strong markers of Th17 polarization [17]. Additionally, a previous study analyzing the innate immunity during acute CHIKV infection revealed a marked increase in the levels of IL-6 [11], which contributed to the Th17 cells’ differentiation. The results presented here align with previous studies highlighting the role of the Th17 response during viral infections [30,31,32]. In addition, Th17 cells are also known for their roles in inflammation and tissue damage and could be related to the severe joint and muscle pain characteristic of Chikungunya fever [33,34,35]. 

In contrast to the stimulation of inflammatory response-related markers, our findings showed a marked decrease in TGF-β levels. The Treg cell population is defined by the expression of FoxP3; it produces IL-10 and TGF-β and plays a crucial role in maintaining immune tolerance and preventing autoimmune responses [36]. Besides the increased expression of IL-10, the TGF-β expression was remarkably decreased within the CHIKV-positive individuals; these results might evidence a dichotomy in the regulatory cytokine response during acute CHIKV infection. IL-10 is generally anti-inflammatory, produced by several immune cell types, such as T helper lymphocytes, monocytes/macrophages, and dendritic cells [37,38], and its upregulation represents a form of negative feedback to control excessive inflammation [38,39]. Conversely, the downregulation of TGF-β, a crucial cytokine for Treg functionality [40], can suggest a decrease in Treg differentiation alongside the reduction in the viral load. This is further supported by the negative correlation between the CT values for the viral load and the FoxP3 and TGF-β expression found in our study, suggesting that Treg function might play a negative role in controlling viral replication. The correlation analysis showing a positive relationship between the CT values for the CHIKV viral load and Tbet expression highlights the immunological dynamics during CHIKV infection that are essential to control viral replication. A previous study has shown, through multiparametric flow cytometry, that CHIKV-positive patients displayed a reduced frequency of Treg cells and a marked decrease in the expression of Treg-related markers, such as TGF-β [41], underscoring the regulatory response profile during CHIKV infection. 

The contrasting immune responses suggest a complex balance between pro-inflammatory and regulatory mechanisms, where the reduction in TGF-β might reflect compromised Treg function, further contributing to uncontrolled viral replication. These findings underscore the importance of understanding the relationship between transcription factor expression and the viral load, not only in predicting the disease severity but also in informing the development of targeted therapeutic interventions. Further investigation into how these transcription factors correlate with clinical symptoms and disease progression could provide deeper insights into their roles in CHIKV pathogenesis and their potential as biomarkers.

While our study provides valuable insights into the adaptive immune response during acute CHIKV infection, our cross-sectional design limits our ability to draw conclusions about the temporal dynamics of the immune response during CHIKV infection. In this sense, longitudinal studies are essential to map the progression of immune responses from acute infection to recovery, providing insights into the mechanisms underlying persistent symptoms and potential chronic manifestations. Additionally, exploring the genetic and environmental factors that influence individual variations in the immune response could help to identify biomarkers for disease severity and targets for therapeutic approaches.

## 5. Conclusions

In summary, our study sheds light on the intricate immune responses during acute CHIKV infection, characterized by a mixed Th1, Th2, Th17, and Treg response profile. These results emphasize the complex interplay between different adaptive immune responses and how they may contribute to the pathogenesis of Chikungunya fever. Understanding the dynamics is crucial in developing targeted therapies that can modulate the immune response, reduce inflammation, and alleviate symptoms in CHIKV patients. Future research should focus on longitudinal studies to further elucidate the temporal changes in the immune responses and their correlations with disease severity and recovery.

## Figures and Tables

**Table 1:** Probes and primers used for CHIKV detection by qRT-PCR.

Probe or Primer	Sequence (5′-3′)	Position in the Genome
Prob VCHIK 6919P	AGGTACGCGCTTCAAGTTCGGCG	6919–6941
VCHIK 6856F (+)	TCACTCCCTGTTGGACTTGATAGA	6856–6879
VCHIK 6981R (−)	TTGACGAACAGAGTTAGGAACATACC	6981–6956

**Table 2:** Sequences of primers used in qRT-PCR reactions.

Primer	Forward	Reverse	MT ^1^
β-actin	TGACTCAGGATTTAAAAACTGGAA	GCCACATTGTGAACTTTGGG	56.5
GATA-3	GTCCCTTTCGACTTGCATTT	TATCCATCGCGTTTAGGCTTC	56.9
T-bet	AATGCCGAGATTACTCAGCTG	AAAGTTCTCCCGGAATCCTT	56.9
RORγT	TGACCAGATTGTGCTTCTCAAA	TCCTAACCAGCACCACTTCCAT	58.2
PU.1	AGAAGAAGATCCGCCTGTACCA	GTGCTTGGACGAGAACTGGAA	60.0
AHR	CAGCGTCAGTTACCTGAGAGCCAAG	CGCAAACAAAGCCAACTGAGGTGGAAG	65.1
FoxP3	AGGAAAGGAGGATGGACGAA	AGGCAAGACAGTGGAAACCT	57.8
STAT3	CTGTTTCAGAGGCTAGGTTGTT	AGGTGAGGGACCTTTAGACAC	59.1
IL-4	AACAGCCTCACAGAGCAGAAGAC	GTGTTCTTGGAGGCAGCAAAG	61.0
IL-9	CTTCTGGCCATGGTCCTTAC	CATGGTCTGGTGCAGTTGTC	59.8
IL-10	AGATCTCCGAGATGCCTTCA	ATTCTTCACCTGCTCCACGG	58.8
IL-17	CAATGACCTGGAAATACCAA	TGAAGGCATGTGAAATCGAGA	54.9
IL-22	TTCCAGCAGCCCTATATCACC	GCTCACTCATACTGACTCCGTG	60.9
IFN-γ	ATGCAGAGCCAAATTGTCTCC	AGGCAGGACAACCATTACTGG	59.0
TGF-β	ATTGAGGGCTTTCGCCTTAG	TGTGTTATCCCTGCTGTCACAG	58.9

^1^ Melting Temperature.

## Data Availability

Dataset available on request from the authors.

## Supplementary Materials

The following supporting information can be downloaded at: https://www.mdpi.com/article/10.3390/v16091387/s1, Figure S1: Correlation analysis between the viral load and mRNA expression level for immune-related markers.
