# Supplementary material for: Acute Chikungunya Virus Infection Triggers a Diverse Range of T Helper Lymphocyte Profiles
Source: Viruses. 2024 Aug 30;16(9):1387. doi: 10.3390/v16091387 (PMC11437511; doi:10.3390/v16091387)
Supplement: Supplementary file 1 [file viruses-16-01387-s001.zip › viruses-3135727-supplementary.pdf]

viruses

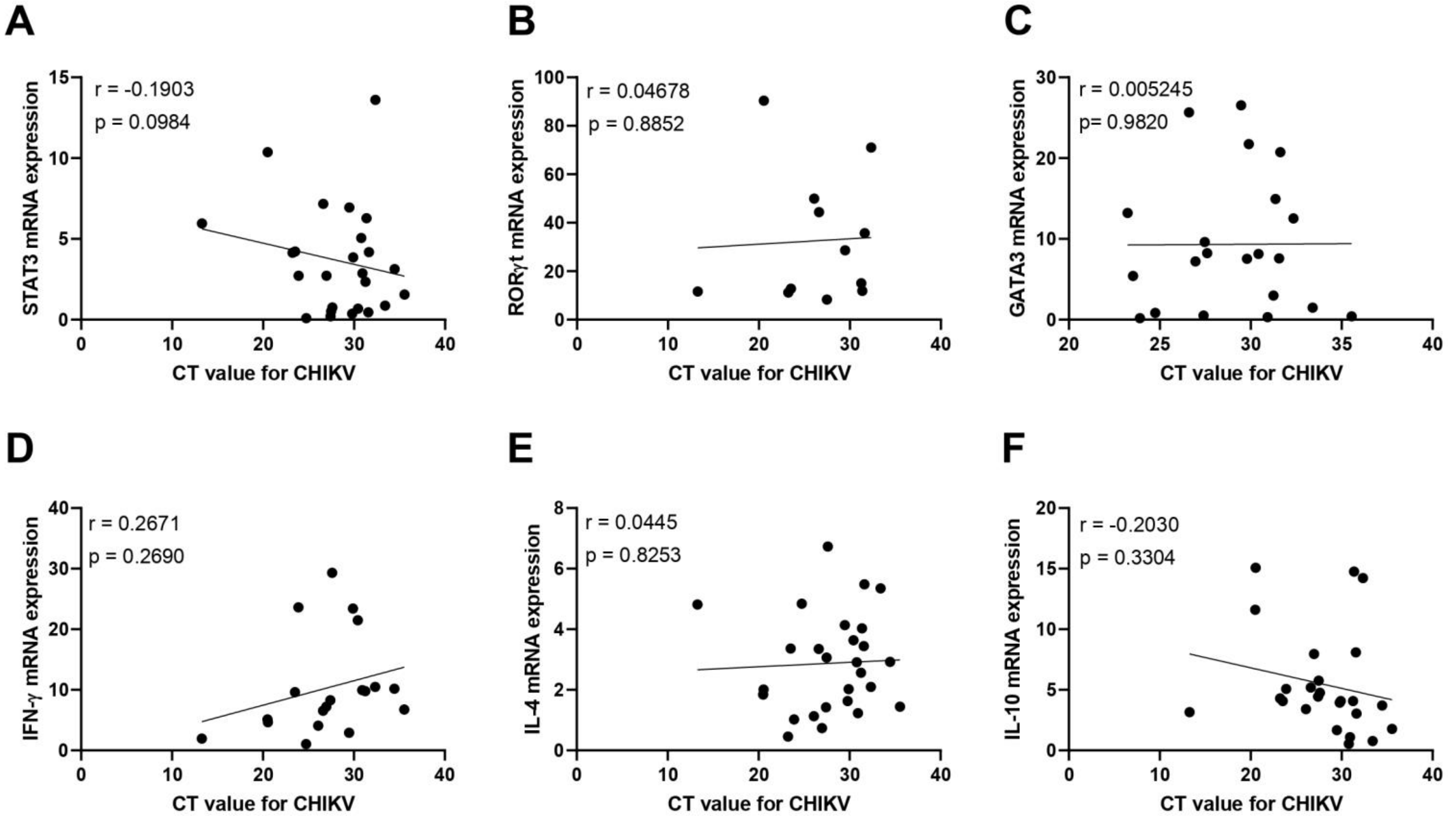

Figure S1. Correlation analysis between the viral load and mRNA expression level for immune-related markers. Correlation analysis between the CT values and mRNA expression for STAT3 (**A**), RORγt (**B**), GATA3 (**C**), IFN-γ (**D**), IL-4 (**E**), and IL-10 (**F**) in patients with acute CHIKV infection, compared to healthy individuals. The analysis was performed using Spearman Correlation test.
